# Recurrent Subclavian Steal Syndrome: A Novel Case of Vasculopathy

**DOI:** 10.7759/cureus.33310

**Published:** 2023-01-03

**Authors:** Daniel F Leach, Daniel M Radwanski, Paramjit Kaur, David D Das, Mamatha Kondapalli

**Affiliations:** 1 Internal Medicine, Southeast Health, Dothan, USA; 2 Radiation Oncology, University of Florida Health, Gainesville, USA; 3 Department of Radiation Oncology, University of Virginia Health, Charlottesville, USA; 4 Transitional Year, Southeast Health Medical Center, Dothan, USA; 5 Internal Medicine, Southeast Health Medical Center, Dothan, USA; 6 Internal Medicine, Medical University of Lublin, Lublin, POL; 7 Hospital Medicine, Southeast Health Medical Center, Dothan, USA

**Keywords:** transcranial doppler ultrasound, digital subtraction angiography(dsa), vertebrobasilar insufficiency (vbi), orthostatic cerebral hypoperfusion syndrome, subclavian steal syndrome

## Abstract

Subclavian steal syndrome (SSS) refers to the phenomenon of retrograde flow in an ipsilateral branch of the subclavian artery due to hemodynamically significant stenosis or occlusion of the ipsilateral proximal subclavian artery. While SSS is usually asymptomatic, it can manifest as vertebrobasilar insufficiency (VBI), ischemia of the affected extremity, or cardiac angina when an internal mammary artery (IMA) is used as a bypass graft. The underlying etiology is most often atherosclerosis but can include Takayasu arteritis, thoracic outlet syndrome, cervical rib, and stenosis secondary to surgical repair of aortic coarctation or tetralogy of Fallot.

There are several case reports describing unique presentations of SSS as well as limited reports of double SSS, where the brachiocephalic steno-occlusive disease causes flow reversal in both the ipsilateral vertebral and carotid arteries. We report herein the first documented case, to our knowledge, of a patient with SSS previously treated with left subclavian artery stenting and left common carotid-subclavian bypass who developed recurrent SSS in conjunction with orthostatic cerebral hypoperfusion syndrome (OCHOS) secondary to severe vasculopathy. She presented with recurrent, paroxysmal vertigo and near-syncope associated with left upper extremity paresthesias that would only abate with sitting in the context of left subclavian artery stent restenosis and occlusion of her left common carotid-subclavian bypass graft. Interestingly, her initial presentation entailed retrograde flow from the left vertebral artery to the left subclavian artery, classic for SSS, but recurrence of her SSS involved retrograde flow from the left common carotid artery to the left subclavian artery, a phenomenon which has also not been described in the literature to our knowledge. As her symptoms of VBI appeared to be triggered by standing and not left arm movement, they were considered to be primarily secondary to OCHOS. Consequently, her primary treatment was to increase salt and fluid intake and thus increase intravascular volume for improved cerebral perfusion as she was not deemed to be a suitable candidate for regrafting of the left subclavian artery.

## Introduction

Subclavian steal syndrome (SSS) was first described in the 1960s with the neurologic association of transient ischemic attacks. Symptomatic SSS is quite rare, and more often found in males over 50 with a 2:1 male-to-female ratio [1]. The incidence and prevalence of SSS are not well defined as many patients remain asymptomatic and are unaware until incidental imaging or diagnostic evaluation is performed. Studies have shown the incidence of extracranial arterial disease due to subclavian occlusion was 17% and approximately 9% exhibited symptoms. The most common complaint of symptomatic SSS is arm claudication during increased activity or exercise due to insufficient blood flow through the stenosed/occluded subclavian vessel [2]. If significant stenosis is present in the subclavian artery, it may result in low pressure distal to the lesion and retrograde flow to ensure adequate blood supply of the affected upper extremity which can manifest as vertebrobasilar insufficiency (VBI) [1,3]. Oftentimes, SSS is suspected after a blood pressure discrepancy is discovered between the upper extremities [3]. In fact, the severity of SSS has been associated with the size of the blood pressure difference between upper limbs being directly proportional; the greater the difference in blood pressure, the greater the severity of SSS [4]. SSS is classified into three grades each based on the territory of blood stolen or severity of the hemodynamic disturbance [1]. SSS is formally diagnosed through ultrasonography and advanced imaging, including computed tomography angiography (CTA), magnetic resonance angiography (MRA), or digital subtraction angiography (DSA). Ultrasound techniques (i.e., transcranial doppler (TCD) ultrasonography) seem to be more sensitive than DSA for screening, but DSA is required for confirmation as not all retrograde vertebral flow elucidated by ultrasonography is truly representative of SSS [1,2]. DSA also provides an opportunity for intervention. The hyperemia-ischemia cuff test often monitored with ultrasound, may uncover occult SSS [1]. Double SSS appears to be exclusive to right-sided circulation due to the brachiocephalic artery giving rise to both the right common carotid and right subclavian arteries, whereas the left common carotid and left subclavian arteries have their own separate origins from the aortic arch (with the exception of anatomical aortic arch variance). This double SSS accounts for less than 2% of all extracranial causes of cerebrovascular insufficiency [5].

## Case presentation

A woman in her late 50s with a past medical history of hypertension, type 2 diabetes mellitus, hyperlipidemia, hypothyroidism, coronary artery disease (CAD) status post-percutaneous coronary intervention, bilateral carotid artery stenosis (CAS), left subclavian artery stenosis with prior SSS, bilateral iliac stenosis with the occlusive disease, peripheral artery disease, myocardial infarction (MI), and ischemic stroke with resolved left-sided weakness presented with episodic near-syncope. The near-syncopal episodes began 2 months prior with up to five episodes daily and were precipitated by standing or ambulation. However, one episode did occur at rest. Each episode would entail lightheadedness followed by blurred vision, left head/neck pounding, left upper extremity paresthesias, generalized tremulousness of the extremities, and then loss of consciousness for 15 seconds if the patient was unable to sit. She reported no postictal state, history of seizure disorder, or witnessed convulsions. She also reported ataxia, palpitations up to 120 beats per minute, and episodic hypertensive crisis with blood pressure up to 180/100, and concomitant, intractable frontotemporal headaches lasting 5 minutes. While she also had a history of migraines, she stated her current headaches were of a different character than the migrainous headaches. Prior to the onset of episodes, the patient was functionally independent and active. She was a former smoker but quit 13 years ago.

Her past surgical history was extensive with 1) CAS treated by bilateral carotid endarterectomy, left common carotid artery stenting, and endarterectomy redo for recurrent disease, and 2) SSS treated by left subclavian artery angioplasty, stenting, and left common carotid-subclavian bypass. Prior to the presentation, the left subclavian artery stent had occluded. According to the family, the patient also had a genetic predisposition to vascular webbing but could not provide a prior diagnosis. Physical examination exhibited bilateral carotid bruits, palpable nonfunctional carotid-subclavian bypass in the left neck, decreased capillary refill, and gait instability. Blood pressure was 150/65 mmHg in the right arm and 83/48 mmHg in the left arm. The patient reported left arm claudication after raising the arm for several seconds.

The basic metabolic panel was significant for an estimated glomerular filtration rate of 50.7, possibly indicative of renal artery stenosis. The hepatic function panel, lipid panel, and complete blood count with differential were unremarkable. High sensitivity troponin was within the normal limits. Coagulation labs were significant for a prothrombin time of 23.3 and an international normalized ratio (INR) of 1.9 although this was in the context of chronic warfarin therapy with an INR goal of 2. Hemoglobin A1c was 5.5%, within the reference range. Thyroid-stimulating hormone was decreased at 0.14 U/ml but free thyroxine was normal at 1.53 ng/dl. Vitamin B12 was elevated at 1287 pg/ml and folate was elevated at >22.3 ng/ml. Syphilis screen exhibited nonreactive treponema palladium antibody. Workup for pheochromocytoma was not performed as the near-syncopal episodes with hypertensive crises were not paroxysmal being triggered by standing.

**Figure 1:**
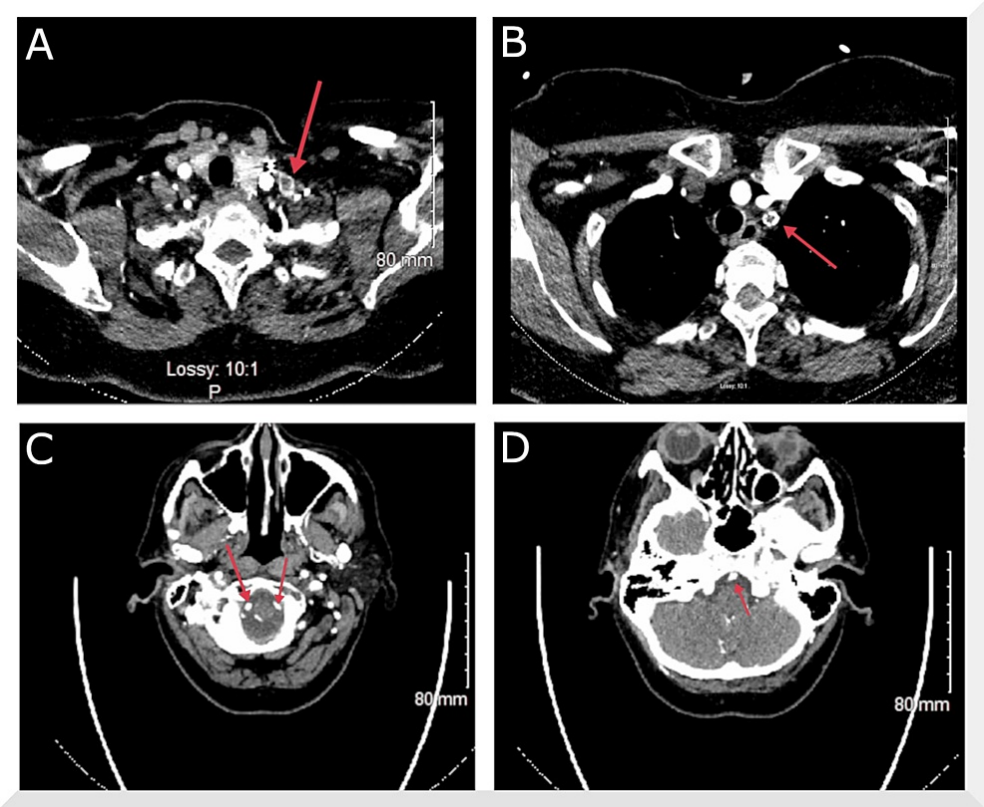
CTA of the head and neck. A) Occlusion of the left common carotid-subclavian artery bypass graft. B) Occlusion of the left subclavian artery stent. C) Patency of the vertebral arteries bilaterally. D) Patency of the basilar artery. CTA: computed tomography angiography

An electrocardiogram (ECG) demonstrated normal sinus rhythm with no ST-segment elevation. Transthoracic echocardiogram (TTE) showed the normal biventricular size and systolic function with an ejection fraction of 50-55%, normal valvular structure and function with only trace mitral regurgitation and trace tricuspid regurgitation, normal great vessels, and normal pericardium. Non-contrast CT of the head showed no acute intracranial abnormality. CTA of the head and neck showed no intracranial large vessel occlusion or high-grade stenosis but revealed an occluded left subclavian artery stent, occlusion of the left common carotid-subclavian bypass, and right brachiocephalic stenosis. However, there was a visualization of bilateral vertebral arteries all the way from the origin and a widely patent basilar artery (Figure 1).

**Figure 2:**
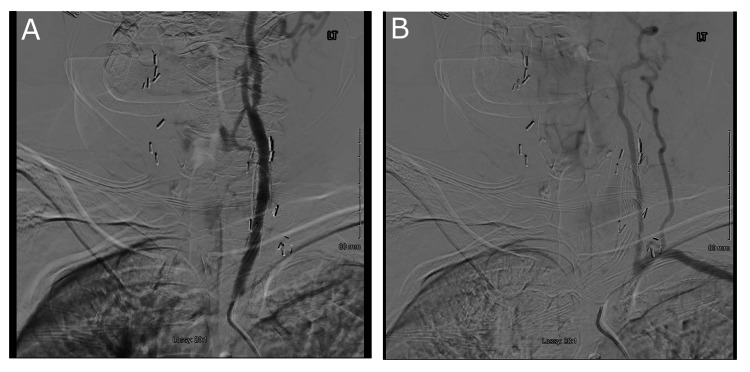
Carotid DSA. A) Fluoroscopic dye injection into the left common carotid artery. B) Transmission of fluoroscopic dye to the left subclavian and left vertebral arteries, visualized as opacification of the vessels, indicating retrograde flow from the left common carotid artery. DSA: digital subtraction angiography

Additionally, there was an estimated 60% stenosis in the proximal right internal carotid artery and 50% stenosis in the left internal carotid artery. Non-contrast MRI of the brain showed no hemorrhage, hydrocephalus, mass shift, or restricted diffusion. MRA of the head showed no significant arterial stenosis, occlusion, aneurysm, or vascular malformation. MRA of the neck showed proximal left subclavian artery stent occlusion, occlusion of the left common carotid-subclavian bypass, patent mid-left common carotid stent, and patency of both vertebral arteries. However, the MRI studies were limited by artifacts from endarterectomy clips, metal stents, and bypass grafts. Carotid DSA then demonstrated complete occlusion of the left subclavian artery stent, which could not be recanalized despite multiple attempts, and SSS as indicated by retrograde flow from the left common carotid artery with filling of the left subclavian and left vertebral arteries (Figure 2).

However, it appeared that there were also large extraluminal collateral branches from the left common carotid artery to the left subclavian artery. Additionally, there was mild to moderate stenosis of the brachiocephalic artery, 65% stenosis of the distal right common carotid artery and internal carotid artery origin secondary to ulcerative plaque, and extensive stenting and distension of the left common carotid artery.

The patient was evaluated by vascular surgery which discontinued the chronic warfarin therapy due to a lack of clear indication. The patient was taking 81 mg of aspirin and 10 mg of atorvastatin daily. The atorvastatin was increased to 40 mg, and the patient was started on 75 mg of clopidogrel daily for dual antiplatelet therapy (DAPT). As no acute vascular surgical intervention was indicated during her admission, she was referred to a tertiary care center for a possible redo of the left common carotid-subclavian bypass for symptomatic SSS.

Orthostatic TCD ultrasonography showed decreased velocities in the right anterior cerebral, right vertebral, and basilar arteries consistent with a drop in blood pressure but showed anterograde vertebral flow. As such, a tilt table test was performed, which replicated the patient’s symptoms, induced tachycardia up to 138 beats/min, and showed mild supine hypertension indicative of severe vasoconstrictor impairment. In totality, these findings were interpreted as orthostatic cerebral hypoperfusion syndrome (OCHOS). As such, the patient was instructed to decrease the dosages of her blood pressure medications and increase salt/fluid intake. It was determined that a redo of the left common carotid-subclavian bypass would entail excessive risk due to the presence of multiple medical comorbidities. Additionally, her left common carotid stent and left endarterectomy were patent.

## Discussion

Syncope can be of vasovagal, or neurocardiogenic, origin where triggering stimuli such as the sight of blood or emotional distress rapidly increase vagal tone which results in bradycardia, vasodilation, and precipitous hypotension that overrides autoregulatory baroreceptor mechanisms. Syncope can also be situational, triggered by parasympathetic processes such as micturition, gastrointestinal stimulation (i.e., swallowing, defecation), coughing, and laughing, which increase vagal tone and decrease peripheral sympathetic tone by mechanisms similar to those in vasovagal syncope, culminating in acute cerebrovascular insufficiency. Orthostatic hypotension secondary to dysautonomia or hypovolemia may cause syncope as well. These processes were considered unlikely in our patient as she would have multiple near-syncopal episodes per day in the absence of specific triggers, one even while sitting, which were accompanied by hypertensive urgency. Furthermore, the orthostatic TCD and carotid ultrasounds coupled with the tilt table test evidenced OCHOS, which entails abnormal orthostatic cerebral blood flow in the absence of systemic orthostatic hypotension. Syncope due to metabolic conditions was ruled out by normal bloodwork.

Cardiogenic syncope was considered where arrhythmias, valvular obstruction, or ischemic disorders precipitate low cardiac output and consequent cerebral hypoperfusion. However, initial ECG and troponins were unremarkable. Additionally, continuous telemetry did not reveal any arrhythmias. The patient underwent TTE which effectively ruled out structural abnormalities, most notably hemodynamically significant aortic stenosis. Carotid DSA demonstrated recurrent SSS most likely secondary to steno-occlusive disease of the left subclavian artery and left common carotid-subclavian bypass graft as evidenced by the CTA and MRA studies. However, the collaterals from the left common carotid artery to the left subclavian artery may have been contributory to the observed retrograde filling of the left subclavian artery.

The diagnosis of SSS was based on the three following criteria: 1) evidence of subclavian steno-occlusion, 2) retrograde flow of the left common carotid artery into the left subclavian artery, and 3) patency of both vertebral and basilar arteries when considering the CTA, MRA, and DSA studies in totality [6]. Blood pressure discrepancy between the left and right arms as well as left arm claudication with activity were supportive of hemodynamically significant subclavian steno-occlusive disease. However, the orthostatic TCD and carotid ultrasounds did not show retrograde vertebral flow (although prior studies 7 years ago did). As the patient’s near-syncopal episodes were not triggered by left arm activity but rather by standing, the etiology of these episodes by SSS was questionable. While the patient clearly exhibited recurrent SSS secondary to vasculopathy, the diagnosis of OCHOS may better explain the near-syncopal episodes associated with concurrent systemic hypertension and tachycardia as they were triggered by standing and not left arm activity. This is further indicative of vasculopathy when considering that the tilt table test showed severe vasoconstrictor impairment. It is unclear why the patient had such significant vasculopathy with multiple clinical manifestations and recurrent SSS. The most likely rationale is poorly optimized medical therapy as she was not on DAPT or a high-intensity statin after multiple angioplasty and stenting procedures. Poor lifestyle may have had an effect, especially considering a prior history of smoking. Additionally, she had a family history of unspecified vascular webbing which appears to accelerate atherosclerosis and vascular events [7].

**Figure 3:**
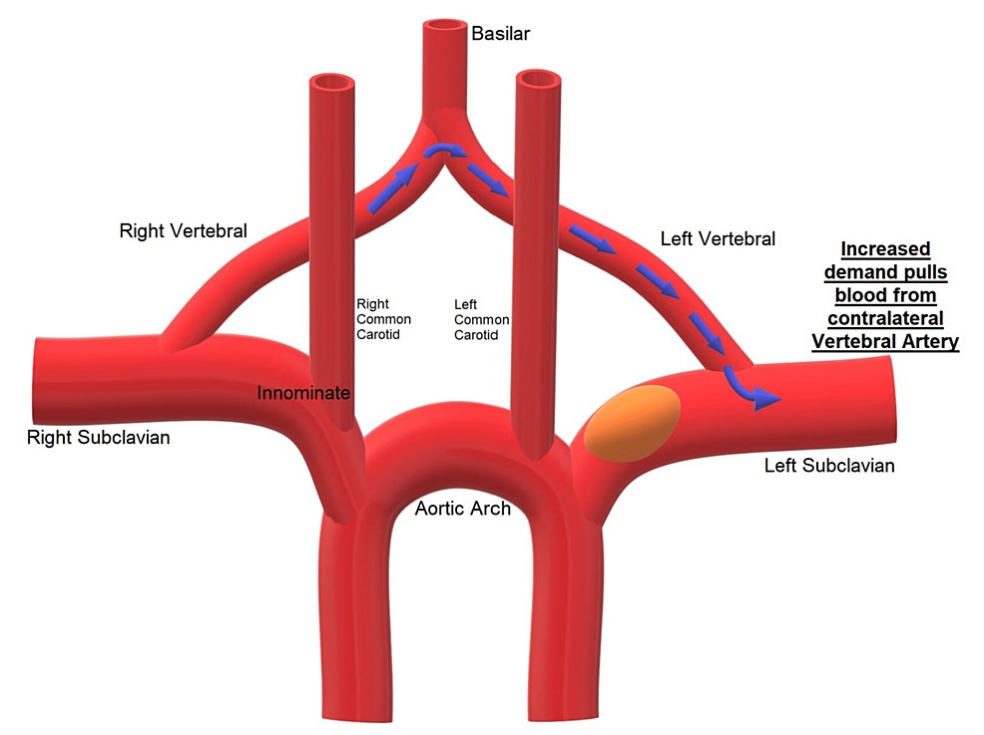
Mechanism of posterior circulation insufficiency. Decreased blood pressure in the left subclavian artery due to steno-occlusive disease coupled with increased demand causes retrograde flow from the right vertebral artery through the vertebrobasilar junction to the left vertebral artery [5].

Syncope due to neurogenic causes was also considered. However, posterior circulation stroke was low on the differential due to normal head CT/CTA, which showed no hemorrhage, ischemic clot, or vascular abnormalities. It is possible that posterior circulation insufficiency was present in the form of retrograde flow to the left vertebral artery through the vertebrobasilar junction, but a catheter-based diagnostic cervicocerebral angiogram was not performed due to the diagnosis of SSS and invasiveness of the procedure (Figure 3) [5].

Furthermore, carotid ultrasonography showed anterograde flow in the external carotid arteries and orthostatic TCD ultrasound showed anterograde flow in the vertebral arteries.

Our case presentation of recurrent SSS secondary to atherosclerotic etiology coupled with OCHOS is unique and, to date, has not been documented in the literature. Only one other case of recurrent SSS has been described in the literature, but due to Takayasu arteritis, a nonatherosclerotic vasculitis [8]. The predominant etiology of SSS is an athero-occlusive disease for which our patient had many metabolic risk factors (including smoking history) and prior clinical sequelae, namely ischemic stroke and MI. Despite multiple angioplasty, stenting, and bypass procedures, she developed symptomatic VBI and was ultimately diagnosed with recurrent SSS. Regarding her subclavian stenotic disease, patients with significant stenosis have a recurrence rate of 10% after stenting which is decreased to 5% if stenting is coupled with angioplasty [9]. As our patient was initially treated with endovascular intervention, she may have been predisposed to restenosis.

Atherosclerosis is by and large the most common cause of subclavian stenosis, and consequently, steal syndromes [1,2]. Numerous case reports have described atherosclerotic SSS [10]. The primary intervention in such cases is percutaneous transluminal angioplasty with stenting [11,12]. Rarely, intravascular procedures can actually precipitate iatrogenic SSS due to prothrombotic vascular injury [13]. When coronary artery bypass procedures utilize an internal mammary artery (IMA) attached to a stenosed subclavian artery, it may “steal” blood from the IMA graft causing divertive retrograde flow in the graft due to increased demand in the affected limb during exertion. This can cause myocardial ischemia manifesting as unstable angina, MI, or sudden cardiac death [2]. Other rare precipitants of SSS include aneurysms [14], dissection [15], and aberrant cardiovascular malformations [16]. An even more rare cause of SSS is Takayasu arteritis which has a wide range of presentations, including recurrent and bilateral SSS [8,17]. While SSS is typically left-sided due to the acute angle of the left subclavian artery, which predisposes endothelial damage and atherosclerosis from increased turbulent fluid dynamics, right-sided SSS has also been described [11,15]. Furthermore, there are SSS mimics secondary to brachiocephalic artery occlusion [5,18], termed “double steal”, and vertebral artery stenosis [19] which primarily presents as VBI similar to SSS. Incredibly, bilateral SSS secondary to simultaneous brachiocephalic and left subclavian artery occlusion has also been reported [20].

## Conclusions

SSS is typically characterized by retrograde flow in an ipsilateral branch of the subclavian artery secondary to proximal steno-occlusive disease. Most patients are asymptomatic but may present with vertebrobasilar or brachial insufficiency manifesting as paroxysmal vertigo or arm claudication, respectively. Atherosclerosis of the subclavian artery is the primary culprit but numerous other causes have been described, including Takayasu arteritis. As such, percutaneous transluminal angioplasty and stenting is the preferred treatment modality with bypass grafting of the subclavian artery performed in refractory cases.

In this report, we describe a case of recurrent SSS in which retrograde flow from the left common carotid artery occurred due to occlusion of the left subclavian artery. Furthermore, recurrence was precipitated by severe vasculopathy. Both of these phenomena have not been described in the literature to our knowledge. The co-presentation of SSS with OCHOS exacerbated the VBI symptoms experienced by the patient. Consequently, clinicians should be aware that recurrence of SSS can occur in patients with severe atherosclerosis despite extensive intervention.
